# The labor market outcomes of bilinguals in the United States: Accumulation and returns effects

**DOI:** 10.1371/journal.pone.0287711

**Published:** 2023-06-29

**Authors:** Olga Churkina, Luísa Nazareno, Matteo Zullo

**Affiliations:** 1 Andrew Young School of Policy Studies, Georgia State University, Atlanta, Georgia, United States of America; 2 School of Public Policy, Georgia Institute of Technology, Atlanta, Georgia, United States of America; Free University of Bozen-Bolzano, ITALY

## Abstract

At least half of the world population is bilingual, but lifetime financial gains from early exposure to multiple languages are largely unknown. In this study, we analyze individual earnings of bilinguals in the US using 15 years of Census data and an augmented wage model, accounting for cognitive, manual, and interpersonal skills derived from O*NET job task descriptors via sparse principal component method. Using unconditional quantile regression, we find evidence that language skills mostly benefit individuals at the lower end of the earnings distribution. While our analysis does not establish causality, it underscores the potential of early language acquisition to mitigate income inequality by enhancing the employment prospects of low-income individuals. We also highlight the favorable cost-benefit ratio of language acquisition in childhood, when learners face no monetary opportunity costs and can achieve greater levels of fluency.

## Introduction

Multilingualism is the ability to switch between two or more languages at the same or about the same level of proficiency [1]. A significant portion of the global population possesses this skill, with estimates suggesting that over half of individuals are bilingual [2], and globalization brings an increasingly multilingual workforce to workplaces. Furthermore, individuals raised as multilingual pledge virtually no effort into acquiring language skills and face no monetary opportunity costs when learning a second language [3, 4].

Despite all of this, studies conducted in the US have been inconclusive regarding benefits from multilingual skills [5, 6]. These studies often abstract language skills from the iterative process of human capital development [4, 7] by pooling together multilinguals and language learners [8] and overlooking the different returns to languages for individuals with varying levels of non-language skills.

Definitions of ‘multilingualism’ might be weaker or stronger, discrete or modular [9]. Our identification strategy builds on a strong definition of bilingualism in line with Bloomfield’s (1935) [1]. The word ‘bilingual’ applies to all individuals having native or mother tongue proficiency in two languages (hereinafter, L1 and L2). Scholars agree that the age of first exposure determines the maximum level of L2 fluency that the average learner will attain [10, 11], regardless of whether critical periods for language acquisition exist [10, 12, 13]. In the present study, individuals who learned a second language in their early childhood are referred to as ‘bilinguals,’ and those who learned a second language at a later stage in life are referred to as ‘late learners.’ The ‘monolingual’ category is reserved for workers who declare native fluency in only one language (i.e., English).

Our research contributes to the broader literature on the technology of skill formation ranging across the social sciences. We use worker records from a pooled cross-section of 2005–2019 American Community Survey (ACS) data expanded with job characteristics from the Occupational Information Network (O*NET). Our augmented wage regressions allow separating the effects of second language skills from the effects of non-language skills on individual earnings. Furthermore, using regression decomposition, we are able to identify the differential impact of second language skills across the earnings distribution.

The study differs from prior contributions to the economic analysis of language in both its depth and breadth. Not only it considers a broader skill bundle and identifies dynamic complementarities between different types of skills [4, 14], it also departs from studies that focus on the English language premium in non-English speaking countries [8, 15], and studies on the majority language premium [16–18]. It also advances the literature by expanding the scope beyond bilingual individuals to include late learners, and by observing differences across the earnings distribution. The broad focus of our research places it at the intersection of labor and migration economics, linguistics, sociology, and human development studies begetting the interest of a diverse scholarly and policymaker community. While our analysis contributes methodological and empirical refinements to the literature, it does not assess causality between the variables under investigation.

Our main finding points to substantial distributional differences in the impact of L2 skills on earnings between bilinguals and late learners, on the one hand, and monolingual English speakers, on the other hand. The predicted earnings gap between bilinguals and late learners relative to monolinguals is largest at the median of the distribution and becomes slightly positive at the bottom of the distribution. This could suggest that distributional differences underscore competitive advantages from language skills in industries with relatively undifferentiated labor inputs.

Furthermore, we refrain from attributing gains from bilingualism to enhanced cognition. Theories of human development [19] have suggested that being raised as bilingual improves executive control functions and results in increased cognitive ability. The claim would require that the unobserved component of the monolingual-bilingual wage gap (i.e., returns effect) dominates the observed component (i.e., endowments effect). However, empirical findings indicate that the returns and endowments effects partake almost equally in explaining the gap.

The main limitation to our findings is generalizability to economies other than the US, which has been popularly characterized as a ‘graveyard of languages’ due to having a dominant majority language. It is possible that the returns on second language skills are higher in less majority language-dominated countries. Another limitation is that our financial analysis of language skills might leave out non-monetary benefits from being raised as bilingual, such as improvements in health and emotional regulation [20]. These factors must be considered for a broader understanding of bilingualism.

### Language markets

Language acquisition is considered a form of household investment [21] studied by language economics [3, 22, 23]. According to economic theory, the price of language skills is determined by market demands: the higher the demand for language skills, the larger the financial gains they attract ‘at both the low and higher ends of the skill spectrum’ [24, p.2]. When particular L2 skills are high in-demand, speakers of that language gain access to a wider pool of jobs and/or have the ability to specialize in tasks that involve language skills within their professions [25].

For language skills to partake in individual earnings, it is necessary for the the economy to carry out some of its functions in languages other than the majority language [26]. Unfortunately, the size of the US multilingual job market has not been properly quantified, with the notable exception of a 2005 article by Saiz and Zoido [27]. In the article, the authors parse through job listings in the Monster.com and CareerBuilder.com portals finding out that only three percent of job openings reserved for college graduates required second language skills.

Language premia might be transmitted via economies of scope [28]. In particular, the use of translations becomes an option at lower levels of L2 proficiency of workers or when the technical requirements of a job are low enough to void the need for specialized skills. This can be seen in the healthcare industry, where it is more efficient for healthcare providers to hire bilingual nurses. This is because by having someone who is bilingual, there is no need for an interpreter to facilitate communication between English-speaking nurses and Spanish-speaking patients. This allows for smoother communication and avoids disruptions [28].

Due to economies of scope, bilingual individuals may earn more than monolingual workers in the same industry and profession (as detailed in the Supporting Information file—S1 File). However, this advantage does not extend to bilinguals who enter the primary monolingual job market. In such cases, language skills may become ‘idle’ when the optimal allocation of a bilingual worker is a monolingual job, such as in technical occupations that do not require language proficiency. Therefore, the impact of language skills on earnings is dependent on the specific non-language skills of workers and the market demand for different languages.

### Skill formation

Research on the development of the bilingual brain is longstanding and has been advanced by the availability of neuroimaging in the last two decades [29, 30]. Individuals raised as bilinguals learn to switch between languages and select vocabulary from two or more conflicting sources. While English-Spanish kids to whom an apple is presented would have to suppress one between the word ‘apple’ and ‘manzana,’ English monolinguals do not need to suppress any input. In support of the hypothesis that bilingualism fosters the development of executive control capabilities (i.e., the behaviors required to plan and achieve goals), controlled experiments have revealed that bilinguals outperform monolinguals on tasks that require inhibitory control and selective attention, supporting the hypothesis that bilingualism might enhance the development of cognitive control abilities [19, 20].

Neuroimages have revealed that languages are lateralized in the left hemisphere of the human brain. However, the age of exposure to a second language appears to affect the degree of lateralization. A sequence of studies on Chinese-English bilinguals [29, 30] has shown that the brains of bilinguals differ from the brains of Chinese monolinguals who learned English as adults. Early exposure to both languages leads to the bilingual brain lateralizing the two languages in separate parts of the brain, while individuals who were raised as Chinese monolinguals did not re-lateralize when encoding English. This lack of re-lateralization possibly explains the permanence of native Chinese grammar patterns and intonations among late learners.

Early language acquisition might affect the accumulation of cognitive skills. This argument supports the differential brain development hypothesis pointing out the different accumulation of cognitive skills among bilinguals and monolinguals. Research suggests that the development of human capital is path-dependent, meaning that it builds upon itself over time [4, 7]. Additionally, there is evidence of a dynamic link between non-cognitive skills and cognitive skills later in life, but not in a tangible or clear way in the other direction [14]. These findings suggest that bilinguals might have developed stronger cognitive skillsets that could indirectly affect their earnings. If future studies were to show that cognitive skills affect non-cognitive skills at later times, the findings of our article would still hold true as they test a direct implication of the assumption that non-cognitive skills affect cognitive skills. However, the theoretical framework developed in the S1 File would need to be revised to reflect this new understanding.

### Skilled migration

The debate on immigrant self-selection is established in the skilled migration literature. This literature has identified positive self-selection as a cause for later-life catch-up of immigrant earnings with native workers [31–34]. Additionally, it has been argued that a lack of language capital might cause specialization of immigrant workers in high-paying technical industries [35]. To indirectly control for unobservable heterogeneity potentially driving financial outcomes of bilinguals, the current study includes a group of late language learners in the main analysis.

It is important to note that immigrant status might proxy for negative effects from socioeconomic and ethnic disadvantage [25, 36–38] or lack of competence in the host language [18]. Even though late learners occasionally achieve levels of L2 fluency comparable to that of bilinguals [39], they most often do not. Immigrant late learners also bear high entry costs in the form of travel expenses, cultural adjustment, and lack of support systems. Therefore, bilinguals and late learners who share the same first language should not experience different levels of discrimination based on when they entered the US. Any additional benefits or reduced disadvantages estimated for bilinguals can be understood as being separate from any potential discrimination or bias.

In sum, the inclusion of the late learner group is a meaningful sensitivity check for language effects. If positive self-selection compensates for the disadvantages of limited proficiency in English, it is expected that the late learner group will have better outcomes, on average, compared to bilinguals.

### Estimates review

Research on language and earnings has been conducted in the US, Canada, Germany, Australia, India, and Israel. There is broad consensus that lack of proficiency in the host language worsens the job market prospects of immigrants to the US [16] and to virtually any world countries [15, 18, 40].

The distance between the origin and the host languages determines the size of the language barrier [41–43]. Thus, it has been pointed out that ‘few other investments that an immigrant could undertake would yield such a healthy monetary return’ [16, p.43] and that less educated and less experienced workers tend to suffer the most from incomplete linguistic assimilation. Similarly, a seminal article using data from the 1976 Survey of Income and Education [26] found that accounting for host language proficiency turns ethnicity and immigration status into insignificant predictors of the native-immigrant wage gap. The finding suggests that immigration status and ethnicity might not have independent effects on earnings that are not channeled by language. Similar evidence has been gathered for Canada [44], Germany [45, 46], and Switzerland [47], although it can be challenging to separate the effects of language and ethnicity in these studies. A study seeking to explain the fall-off of Spanish-speaking immigrants to the US in the 1980s compared to the previous decade found it hard to discern the language and ethnicity factors of the decline [48], much like other studies [47, 49].

Few articles [8, 17, 50] have reported positive effects from foreign language proficiency for immigrant workers in Europe. However, their definition of language skills does not distinguish between heritage bilinguals and language learners. Furthermore, immigrant workers in Europe might have been positively self-selected, which would result in biased estimates. This fact might explain why the strongest and most positive effects have been found among English-proficient workers employed in higher-paying professions [8]. Élite workers employed in the largest European corporations and public institutions are often recruited from an English-speaking, highly-educated, and mobile workforce not very representative of the heritage bilingual population.

For the US, there is little evidence of a bilingual earnings premium [51, 52] and some evidence of a bilingual earnings disadvantage [6]. This has led to the belief that public support for language instruction must be predicated upon the cultural merits of language learning more so than on its financial returns. However, more recent studies have provided countering evidence [51, 52]. One of the studies [51] uses longitudinal analysis to track the earnings of children of immigrants transitioning from college to work, finding that children raised as bilingual had marginally higher earnings compared to children raised as monolingual. This result aligns with evidence of an L2 premium for children of immigrants to the state of California [52]. While encouraging, these findings must be cautioned because age-earnings functions are highly volatile until early thirties and may not be a reliable indicator of lifetime earnings [53].

Quasi-experimental studies have been conducted on more narrowly defined bilingual populations. These include the ‘Quebec studies’ [54–57] and other European studies [58, 59]. In the 1970s, Quebec spearheaded his ‘Francophone agenda’ to reduce income inequality between English- and French-speaking Québécois and required the use of French language in the workplace. While the intervention closed part of the gap between the two groups [56], it could not reduce the thriving demand for English skills. Hence, it ended up securing windfall benefits to English-fluent Francophones [54] and establishing that between bilinguals and allophones as the main divide in society [57]. An analogous attempt to rally back the Welsh-speaking population, the 1993 Welsh Language Act subjected public servants to the requirement of double Welsh-English proficiency. Similarly to the English-speaking requirement in Quebec, the requirement has failed to revive the Welsh language and only increased the earnings of Welsh speakers. These studies suggest that government efforts to promote specific languages may not be effective in boosting the use of those languages, and may even create unintended benefits for certain groups.

## Data and methods

### Data

Our primary data source is a pooled cross-sectional dataset accumulated from the American Community Survey (ACS) 1-year estimates over the period 2005–2019. ACS is a nationally representative household survey of the US carried out by the US Census Bureau since 2005. Our sample comprises individuals aged 16 and older for whom we have information on employment and income and meeting the criteria for inclusion in one of the groups defined in this section.

The dependent variable throughout the analysis is the logarithm of wage and salary income in 2005 CPI-adjusted dollars accounting for total earnings before taxes received in the prior 12 months. To account for differences in hours worked, we calculated the annual wage and salary income as if the individual had worked for a full year (52 weeks). Additionally, we removed any extreme values or outliers from the wage distribution [60]: the lower threshold was annual earnings of $852 in 1999 adjusted dollars, which is equivalent to the reference $2,000 threshold for year 1979, and we top-coded earnings at the 99.5^*th*^ percentile in each year.

We used two variables from the American Community Survey (ACS) to define language groups: the level of English proficiency and the language spoken at home. People were considered ‘bilingual’ if they spoke a language other than English at home and were either born in the US or migrated to the US before age six [61]. Those who migrated to the US between the ages of 7 and 16 were identified as ‘L2 learners’ or ‘late learners’. The age of first entry into the US is used as a proxy for the age of first exposure to the L2 (i.e., English). We set the age of 16 as a high cutoff for late learners to reduce concerns about self-selection, as migration decisions at that age are likely made by older family members [36, 37, 62]. All bilinguals and late learners who reported low English proficiency (i.e., do not speak English, or do so but not very well) were removed from the sample and so were workers who did not fit into any the three definitions. Lastly, all respondents who were born in the US and spoke English at home were classified as ‘monolingual.’ We acknowledge that some monolingual individuals may have been exposed to other languages later in life, such as through formal education, but this information is not available in the current data. Also, the identification used in this article is supported by prior research [10, 11] and is a good proxy for language proficiency at the aggregate level. Certainly, more narrowly controlled studies may benefit from a coding of language status that does not rely on individual self-report.

Our operationalization of bilingual proficiency uses a strong cutoff validated by studies of language acquisition [13]. In the scope of the current research, a functional bilingual is not a worker who declares competence in two or more languages [8], but a worker whose dual proficiency in the two languages might be qualified as native or near-native. Additionally, we separate the bilingual and late learner populations to observe the relationship between age of exposure to the second language and anticipated financial outcomes [31, 33, 34].

The ACS data imposes some natural limitations to our study that we must acknowledge. Specifically, self-reported language fluency might not accurately reflect actual language proficiency, a constraint faced by similar studies [41–43, 63]. Likewise, the ACS data does not provide information on language skills beyond English proficiency and the language spoken at home. Data availability also imposes constraints on the temporal extension of the study and the robustness tests we wished to implement. For instance, the lack of information on parents’ country of origin does not allow us to distinguish second-generation immigrants among monolinguals—a group that likely shares similarities with bilinguals. Furthermore, parental information would have allowed us to more fully investigate the role of family socioeconomic conditions as mediators for potential returns to language skills.

Our estimation strategy involved several steps. First, we used occupational codes to merge the sample with O*NET job task descriptors and implemented sparse principal component analysis to generate three measures of non-cognitive skill set: cognitive, manual, and interpersonal skills. Second, we adopted a three-way exact matching strategy to reduce the sample, consisting of bilinguals, late learners, and monolinguals, to three sets which are as similar as possible. This strategy preserves most bilinguals and late learners but significantly reduces the monolingual sample. Third, we used unconditional quantile regressions to study the relationship between language and non-language skills on individual earnings across the earnings distribution. The decomposition provides a comprehensive understanding of the relationship between language skills and worker compensations. Lastly, we performed Oaxaca-Blinder decompositions to decompose observed wage gaps into endowments and returns effects. Each of these steps is discussed more fully in the section that follows.

### Sparse principal component analysis

In our study, we aim to separate the impact of language proficiency from other skills on labor market outcomes. To accomplish this, we must devise a method for measuring non-language skills. One commonly utilized method is to utilize educational attainment as a proxy for skill level and is based on the assumptions that employers seek out employees with the necessary skills for a given job, and that individuals acquire those skills through their education. Using education as a proxy for skills is a convenient method due to the availability of this information in various data sources. However, the correspondence of workers’ education and skill requirements of jobs is not always perfect, and workers commonly believe to be either over-or under-qualified for their jobs [64]. Further, skills that are not captured by test scores such as persistence and self-esteem may also play a role in determining earnings [65].

An alternative approach to operationalizing skills is to focus on the skill content of the tasks carried out as a part of a job. Occupational data sources provide this information, typically presenting a series of skills and tasks and scoring them according to ordinal and sometimes interval-level scales. By directly capturing job content, occupational data is naturally superior to educational attainments as a measure of skills performed on the job. Furthermore, occupational data is multi-dimensional and allows for a more detailed examination of different types of skills, beyond what is captured by test scores. Such classifications, however, are not always available for every country or period of analysis, and comparisons across time and space are not always consistent [66].

Non-language skills were operationalized by way of the task descriptors featured in the Occupation Information Network (O*NET). The O*NET is a database sponsored by the U.S. Department of Labor that provides detailed information at the occupation level on various topics related to the content and context of jobs. For each occupation, O*NET specifies 35 skills, ranked according to their importance and level on a 0 to 100 standardized scale. We used a weighted average to convert these measures into a single scale, using Cobb-Douglas weights of 1/3 and 2/3 for level and importance, respectively [67]. We relied on O*NET’s crosswalks to merge its codes with standard occupational classification (SOC 2010) adopted by the Bureau of Census in the American Community Survey (ACS). The process included: (i) translating ONET codes to SOC codes using the ONET crosswalk file, (ii) translating SOC codes to Census codes using the Census crosswalk file, (iii) translating Census codes to IPUMS (COC 2010 harmonized for several years) codes. The final result was a crosswalk that linked O*NET codes to IPUMS occupational codes.

To reduce down the 35 skills acquired from O*NET to a more meaningful subset, we conducted a sparse principal component analysis (SPCA). This process resulted in three continuous skill variables: cognitive, manual, and interpersonal skills (as detailed in the S1 File). S1 Table in the S1 File illustrates the mapping of task descriptors into the reduced set with the three skill components.

### Three-way matching

To avoid inflated statistical significance and reduce bias in the data, we performed exact three-way matching of the three language groups against selected covariates. This is because analyses of large pooled cross-sections of Census data often provide false positives driven by the sample size. Also, regression-based adjustments in the absence of exact covariate matching might fail to block unobservable heterogeneity. The three-way matching algorithm we developed is particularly useful in the presence of imbalanced datasets with over-representation of one group (e.g., monolinguals) and loses generality when groups are closer in size. Indeed, the ACS sample matching our selection criteria comprises roughly 13 million individuals of which 97 percent are monolinguals (see S2 Table in the S1 File for more details). Generalizations of the algorithm to arbitrary three-group exact matching problems are possible but not in the current code infrastructure.

The algorithm preserves as many bilingual-late learner pairs as possible and matches each pair to a monolingual observation. Two parameters are defined in the three-way exact matching algorithm: one sets the maximum number of assignments allowed for the over-represented group (i.e., monolingual); the second sets the maximum size of each triple (i.e., bilingual, late learner, and monolingual). This is to cover cases when multiple matches are available for the over-represented group and cases where multiple matches are available for the under-represented groups.

To construct appropriate counterfactuals out of the members of the monolingual group, we matched on individual demographics (i.e., sex, race, 5-year age groups, marital status, and level of education) and the cohorts of arrival in the US (i.e., the decade of arrival or, for monolinguals, the decade of birth). We limited the number of monolinguals retained for each year to be equal to or less than the combined number of matched bilinguals and late learners.

**Fig 1:**
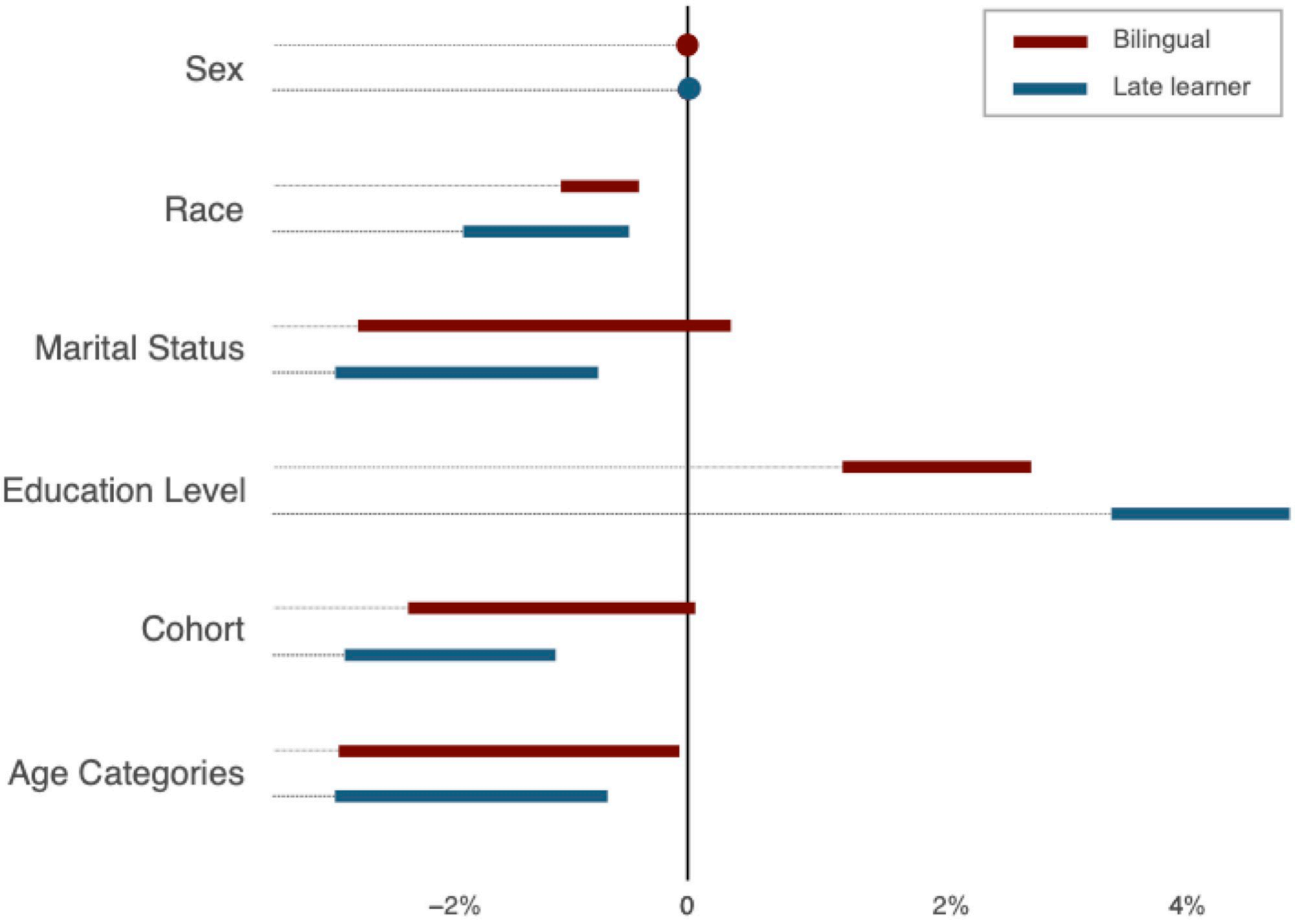
Bias reduction due to the three-way matching. This figure shows standardized percent reduction in bias afforded by three-way matching. The key conditioning variables include sex, race, marital status, education level, cohort, and age 5-year categories. The remaining bias in standardized percent bias is less than 0.5-percent for the ‘bilingual vs. monolingual’ pair and 1-percent for the ‘late learner vs. monolingual’ pair across years. At the same time, the yearly three-way matching eliminated the bias completely in every time period.

Overall, the three-way exact matching procedure yielded near-perfect matching within each year stratum and an overall bias reduction exceeding 99 percent, while preserving about 98 and 95 percent of the original bilingual and late learner populations (see S3 Table in the S1 File for more details). As expected, statistical differences between the matched and initial groups were marginal, although there were some differences between the matched monolingual group and the initial monolingual group. The matched group was closer to the bilingual and late learner groups as a result of the exact-matching procedure excluding observations without common support (see S4 Table in the S1 File for more details). Fig 1 reports a summary of the pre- and post-matching differences using standardized percent bias [68] for each conditioning variable. The standardized percent bias is defined by the formula

$$SB=100\times \frac{{\bar{\text{x}}}_{1}-{\bar{\text{x}}}_{0}}{\sqrt{\frac{1}{2}\left({\text{s}}_{1}^{2}+{\text{s}}_{0}^{2}\right)}}$$

where ${\bar{\text{x}}}_{1}$ and ${\text{s}}_{1}^{2}$ are the mean and the variance of group 1, while ${\bar{\text{x}}}_{0}$ and ${\text{s}}_{0}^{2}$ are the mean and the variance of group 0.

### Econometric model

We estimated variations of an augmented wage model with log-transformed wages as the dependent variable and conditioning on industry, hours worked per week, demographic characteristics, and location and year effects. The regressions are run on three-samples of English monolinguals, bilinguals, and late learners where each triple is an exact match. The focal variables are:

- *Language status*: a categorical variable operationalizing language skills and taking the three values of monolingual, bilingual, or late learner (monolingual is set as the reference group).
- *Cognitive*, *manual*, and *interpersonal* score coefficients: numerical variables operationalizing non-language skills and taking values in the [0, 100] range.

The resulting regression model reads:

$$ln{\text{w}}_{it}=\alpha +\beta_{C}{\text{x}}_{i}^{C}+\beta_{M}{\text{x}}_{i}^{M}+\beta_{I}{\text{x}}_{i}^{I}+\beta_{L}{\text{x}}_{i}^{L}+\beta_{h}{\text{h}}_{it}+\beta_{d}{\text{d}}_{it}+\gamma_{it}+\phi_{it}+\theta_{t}+\varepsilon_{it}$$

where *W*_*it*_ is the logarithm of wages and salary earnings in 2005 CPI-adjusted dollars [69]; ${\text{x}}_{i}^{C},{\text{x}}_{i}^{M},{\text{x}}_{i}^{I}$ are the three non-language capital values for cognitive, manual, and interpersonal skills; ${\text{x}}_{i}^{L}$ is a variable coding language status; **h**_*it*_ is the reported number of weekly work hours; **d**_*it*_ is a vector of demographic controls; *γ*_*it*_ captures industry; *ϕ*_*it*_ is a vector of state-year concentration of non-English speakers; *θ*_*t*_ is a vector of year fixed effects; and *ε*_*jt*_ is an individual error term. Language specialization is operationalized as the year-state location quotients (LQs) for languages spoken. Each LQ is calculated as the ratio of the proportion of non-English language speakers of language *i* in state *s* in year *t* to the equivalent ratio at the national level. LQs larger than 1 reflect a disproportionate concentration of non-English language speakers in a state relative to the country, and LQs lower than 1 reflect a lower concentration.

Any arbitrary level of skills *S* affects individual earnings through a production function $g\left(S\right)=\beta {\text{x}}_{i}^{S}$. We employed the Oaxaca-Blinder decomposition method [70, 71], which is a widely used technique for decomposing the total effect of a variable on earnings into two components: endowments (i.e., characteristics) and returns (i.e., coefficients). The two terms of the decomposition are the mean level of skill endowments and the mean return on each skill. Endowments effects capture the part of the gap attributable to between-group differences in skills, while returns effects capture the part of the gap attributable to between-group differences in the skill rate of returns. Therefore, for the monolingual-bilingual gap and the monolingual-late learner gap, these are given by $\left({\bar{\text{x}}}_{mono}^{S}-{\bar{\text{x}}}_{bi}^{S}\right),\;\left(\beta_{mono}-\beta_{bi}\right)$ and $\left({\bar{\text{x}}}_{mono}^{S}-{\bar{\text{x}}}_{late}^{S}\right),\;\left(\beta_{mono}-\beta_{late}\right)$, respectively. A twofold decomposition [70, 71] decomposes the average predicted earnings gaps $\left({\bar{\hat{\Delta }}}_{mono-bi}\right),\;\left({\bar{\hat{\Delta }}}_{mono-late}\right)$ into the endowments and returns effects. The monolingual-bilingual gap decomposition is therefore given by:

$$\begin{array}{c} {\bar{\hat{\Delta }}}_{mono-bi}=\underset{\text{endowments}}{\underset{︸}{\beta_{mono}\left({\bar{\text{x}}}_{mono}^{S}-{\bar{\text{x}}}_{bi}^{S}\right)}}+\underset{\text{returns}}{\underset{︸}{{\bar{\text{x}}}_{bi}^{S}\left(\beta_{mono}-\beta_{bi}\right)}} \end{array}$$

A twofold decomposition might be conducted at each percentile of the earnings distribution using a recentered influence function (RIF) transform of the dependent variable. The RIF transform is defined as:

$$RIF\left(y,Q_{\tau }\right)=Q_{\tau }+\frac{\tau -I\{y\leq Q_{\tau }\}}{f_{y}\left(Q_{\tau }\right)}$$

where *Q*_*τ*_ is the population *τ*-quantile of the unconditional distribution of *y*, *I*{*} is an indicator function, and *f*_*y*_(*) is the density of the marginal distribution of *y* [72]. Because regression decomposition requires that the average of the conditional differences match unconditional differences in the population, conditional quantile regression may not be used for decomposition. However, use of a RIF transform of the initial conditioning characteristics [72] makes it so that conditional quintiles and unconditional quantiles match and allows decomposing the monolingual-bilingual and monolingual-late learner gaps at any point of the earnings distribution [72] as linear combinations of endowments and returns effects.

The gap in earnings between monolinguals and bilinguals, as represented on the left-hand side of the decomposition, is positive as monolinguals have higher earnings than bilinguals. The total gap is expressed as the algebraic sum of an endowments and returns component, capturing differences in covariate levels and coefficients. When a term is positive, it means that it explains part of the gap, for example that better observable characteristics of monolinguals are responsible for part of their earnings advantage over bilinguals. This also means that equalizing that particular resource would close part of the bilingual earnings gap. To maximize interpretability, we converted differences to dollar values by exponentiating the effect sizes and subtracting the predicted earnings gap.

## Results

### Wage model

Table 1 illustrates differences in log earnings between bilinguals and late learners compared to monolinguals (the reference group), as estimated by our regression models. The errors are clustered at the state level following rejection of the null hypothesis of homoscedasticity of the residual variance via Breusch-Pagan test. Model 1 includes our basic set of control variables: sex, race, marital status, education, a cohort of arrival in the US, location quotients, and year dummies. Subsequent models gradually add additional variables such as weekly hours of work (Model 2), employment industry (Model 3), and non-language skills, including cognitive, manual, and interpersonal skills (Model 4). Comparing the coefficient for bilinguals and late learners across the specifications provides insights into the origins of wage differences.

**Table 1:** Wage regressions with progressive inclusion of variables.

	Model 1	Model 2	Model 3	Model 4
Constant	10.258***	9.448***	9.305***	8.889***
	(0.006)	(0.006)	(0.008)	(0.008)
Bilingual	0.006***	-0.009***	-0.009***	-0.008***
	(0.002)	(0.002)	(0.002)	(0.002)
Late Learner	-0.030***	-0.037***	-0.031***	-0.024***
	(0.002)	(0.002)	(0.002)	(0.002)
Cognitive				0.504***
				(0.003)
Manual				0.197***
				(0.003)
Interpersonal				0.190***
				(0.002)
Hours worked	No	Yes	Yes	Yes
Industry	No	No	Yes	Yes
Demographics	Yes	Yes	Yes	Yes
Location Quotient	Yes	Yes	Yes	Yes
Year	Yes	Yes	Yes	Yes
*Observations*	818,987	818,987	818,987	818,987
*Adjusted R* ^2^	0.359	0.439	0.461	0.491

Notes: 1. ***p < .01; **p < .05; *p < .1; 2. The table presents the results of OLS regressions, in which the dependent variable is the logarithm of wages; 3. The standard errors are clustered at the state level; 4. Demographic characteristics such as sex, race, marital status, level of education, hours worked per week, and the cohort of arrival in the US are included in the analysis; 5. The table shows that industry fixed effects are positive and statistically significant across all specifications; 6. The location quotient is used as a proxy for measuring the concentration of non-English speakers in a given state-year.

Model 1 predicts a small earnings premium for bilinguals of about 0.6 percentage points and a wage penalty of 3 percentage points for late learners compared to monolinguals. However, when controlling for hours worked (i.e., Model 2) and industry (i.e., Model 3), the expected earnings of bilinguals and late learners are 0.9 and 3.1 percentage points less than monolinguals, respectively. This suggests that the bilingual and late learner groups work longer hours and select into higher-paying industries. Thus, after netting out the effect of hours worked and industry, we no longer observe wage premia associated with language skills.

Our preferred specification, Model 4, improves the comparisons by adding control variables for non-language skills. The model confirms that returns to non-language skills are positive and higher for cognitive skills, as previously suggested in literature [73]. However, accounting for non-language skills reduces but does not fully eliminate the wage penalty for bilinguals and late learners, whose expected earnings are 0.8 and 2.4 percentage points lower relative to comparable monolinguals.

Overall, late learners are at a greater disadvantage across all four of our regression model specifications. As such, our results are not consistent with positive self-selection of immigrant workers, nor do they provide any evidence of monetary rewards commanded by language skills.

### Wage gap decomposition

The assumption embedded in our model is that conditional group differences are consistent across the earnings distribution and that returns to skills are also equal. Our expectations are greater recourse to manual skills for those at the lower end of the earnings distribution and greater contribution of cognitive skills among those with larger earnings.

**Fig 2:**
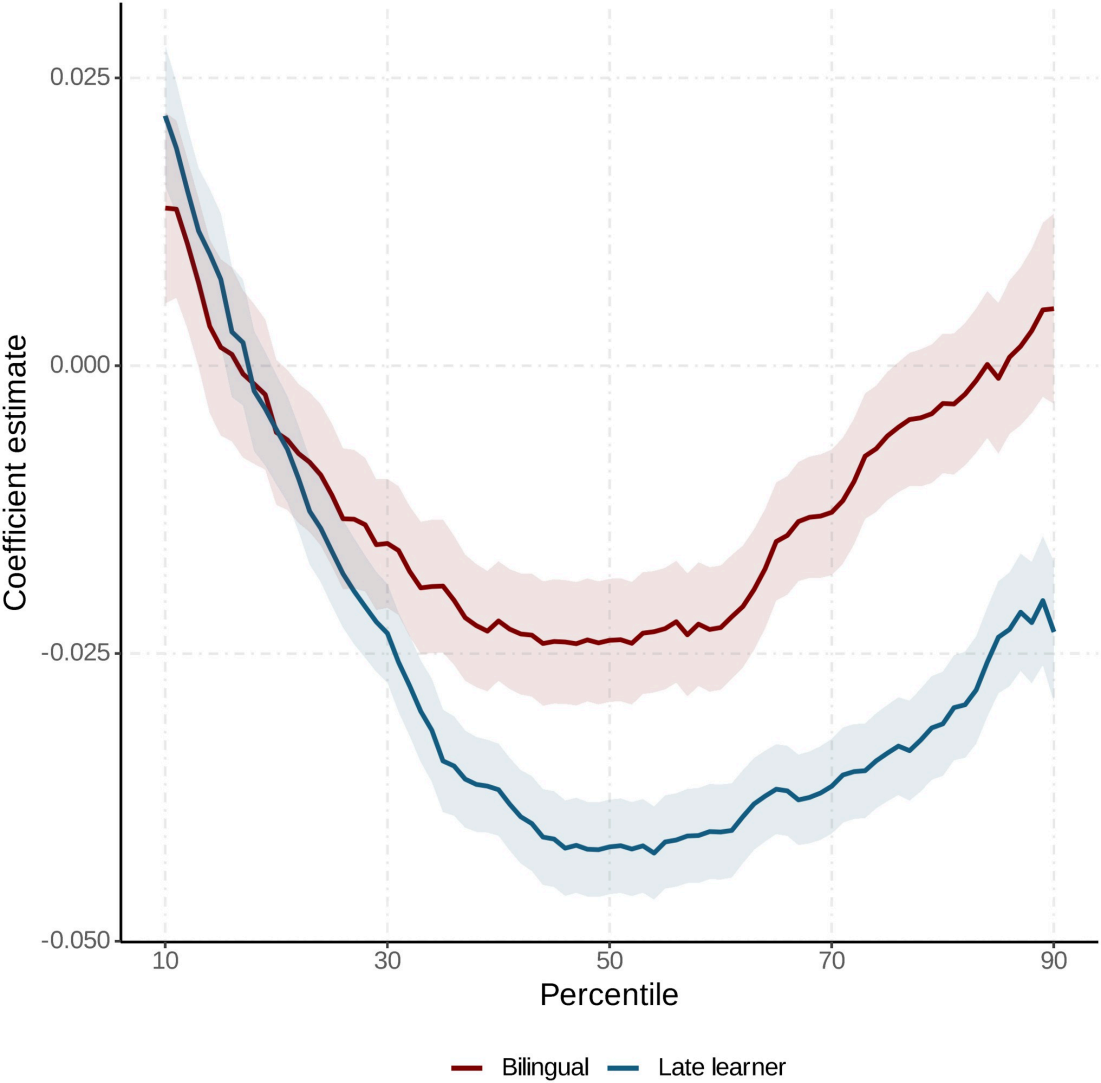
Bilingual and late learner coefficient estimates with 95% confidence intervals. This figure illustrates the coefficient estimates for the bilingual and late learner associations with the logarithm of wage and salary income, represented in percentage points, at each percentile. The coefficients are obtained from unconditional quantile regressions using a recentered influence function (RIF) transformation [72] of the logarithm of income as a function of individual characteristics, skills, industry, and location.

To investigate distributional differences in returns to language skills, we fit the Model 4 specification using RIF transforms of the dependent variable at each percentile [72]. Fig 2 plots the coefficient estimates showing that, for either bilinguals or late learners, earnings penalties are largest about the median of the distribution and shrink thereafter (see S5 Table in the S1 File for more details). At the lower end of the wage distribution, both bilinguals and late learners receive a premium for their language skills, which is consistent with the nature of low-skilled labor markets. In these markets, low-skilled workers supply relatively homogeneous labor, which is easily substitutable, and therefore, language skills have greater selection power and provide a greater competitive advantage, particularly in service industries such as food and entertainment. In comport with Table 1, bilinguals outearn comparable late learners, with no sign of catch-up towards the top end of the distribution. The gap to monolinguals reduces for late learners past the median and disappears for high-income bilinguals.

To identify the specific contributions of skill endowments and returns to skills to wage gaps, we applied twofold regression decomposition [70, 71] and unconditional quantile regressions [72]. This method allowed us to decompose the wage gaps by separately evaluating the impact of skill endowments and returns to skills. The results of this decomposition are directly linked to the econometric model and integral to testing dynamic complementarities between language skills and general cognition. In particular, they allow studyingtesting how language skills relate to differential accumulations of skills among bilinguals.

**Fig 3:**
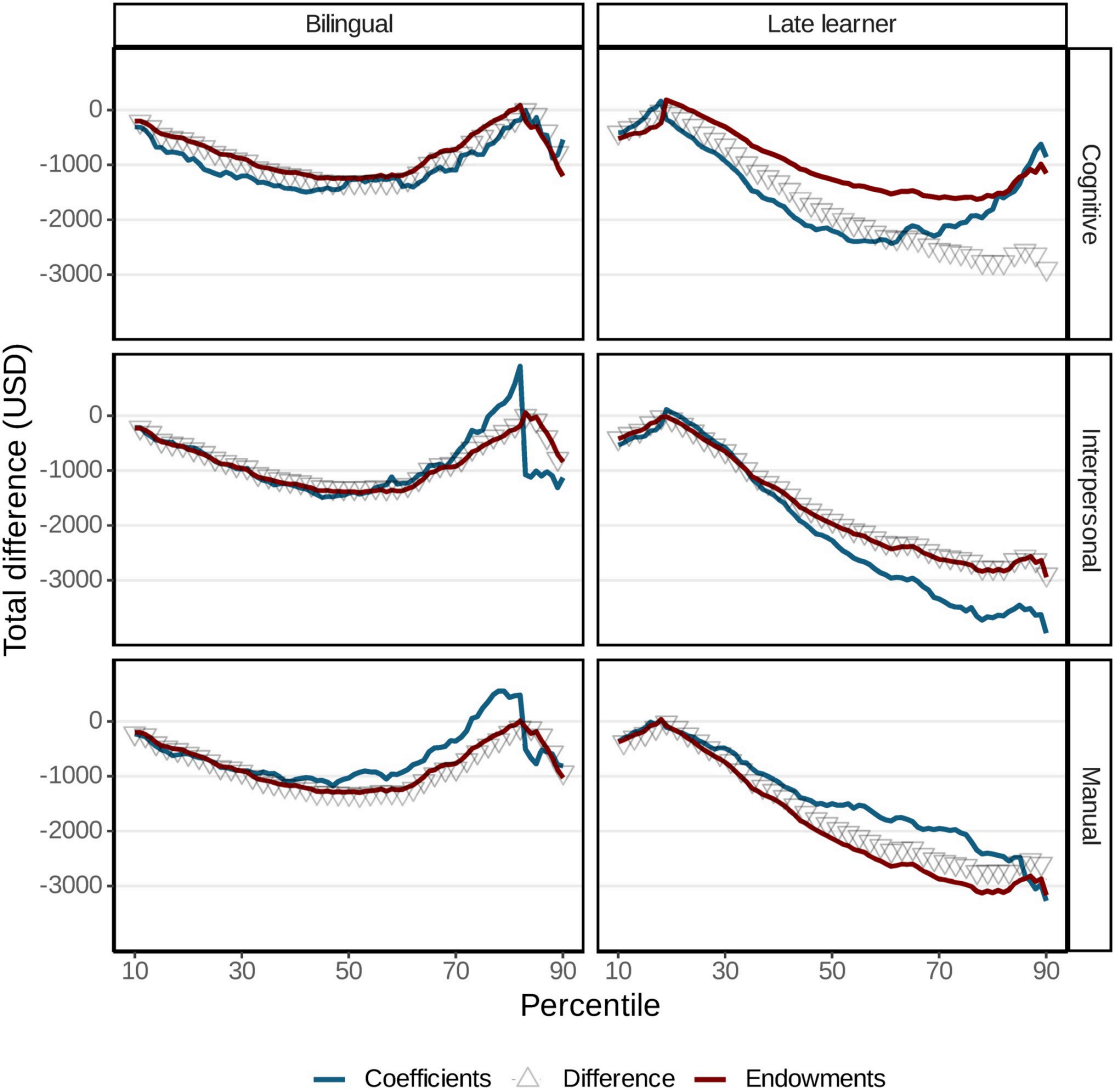
Regression decomposition of wage differences between bilinguals and late language learners. The figure displays the predicted differences in earnings as calculated from independent regression decompositions at each percentile. The earnings gap between bilinguals and late language learners compared to monolinguals is determined by equalizing monolingual skills and returns to skills. The decompositions are carried out using unconditional quantile regressions [72] and are based on a twofold decomposition method [70, 71].

Fig 3 plots average predicted differences in earnings and counterfactual differences when equalizing on skill endowments and returns to skills. The first counterfactual is the case where bilinguals and monolinguals possess the same levels of skills (i.e., cognitive. manual, and interpersonal) while the second counterfactual is the case where bilinguals and monolinguals keep their own skill endowments but receive the same return on those skills.

The decomposition analysis illustrated by Fig 3 yields two main findings. The analysis of the bilingual and late learner groups shows that for bilinguals, the total earnings gap is the largest at the median and follows a *U*-shaped pattern across the earnings distribution. For late learners, the earnings gap follows a downward trend in the earnings distribution. Our analysis shows that below the 80th percentile, equalizing skill endowments or returns would greatly reduce the bilingual-monolingual earnings gap. In fact, if interpersonal and manual returns to skills were the same, the gap would even reverse, meaning that bilinguals would earn more than comparable monolinguals, indicating that the difference in earnings is not due to bilinguals having accumulated greater cognitive skills.

Regarding late learners, the plotted results show that the median late learner would benefit from having the same cognitive skill set as the median monolingual. In fact, their earnings gap would reduce to about half of the average predicted pay difference. Conversely, equalizing returns to skills would leave the gap unchanged. Among top earners, neither equalization would result in equal pay, although the gap would decrease sensibly. For instance, by matching on returns to skills, late learners would make more than what they actually made, and their earnings would be closer to monolinguals. We also observed that late learners would be worse off, if they have the same returns to interpersonal skills as monolinguals.

### Sensitivity checks

To explore variations in the effects, we divided bilinguals and late learners into five smaller language groups that share common cultural features. Breaking down the data into specific groups might be especially useful with regards to the varying levels of demand for different languages. The five groups are: European exclusive of Spanish (i.e., German, Yiddish/Jewish, Dutch, Swedish, Danish, Norwegian, Icelandic, Italian, French, Portuguese, Rumanian, Celtic, Greek, Albanian, Russian, Ukrainian/Ruthenian/Little Russian, Czech, Polish, Slovak, Serbo-Croatian/Yugoslavian/Slavonian, Slovene, Lithuanian, and other Balto-Slavic.), Spanish (mostly Latin Americans), Asian exclusive of Chinese (i.e., Tibetan, Burmese/Lisu/Lolo, Kachin, Thai/Siamese/Lao, Japanese, Korean, Vietnamese, Indonesian, Filipino/Tagalog, and Hawaiian.), Chinese, and Hindi. Each group was independently re-matched using three-way matching technique to eliminate potential bias.

Table 2 presents estimates from our main regression (Model 4 in Table 1). All bilingual and late learner groups, except Europeans, are at a financial disadvantage. Spanish speakers have a larger financial penalty compared to the average reported in the previous section. The Asian population was split into two groups: Chinese and Asian without Chinese. Chinese bilinguals do not earn less than monolinguals, while non-Chinese Asians face a much larger financial penalty. This could be due to the low market demand for non-Chinese Asian languages [28]. Chinese and other Asian late learners are heavily penalized—about 5 and 6 percentage points, respectively—which could be linked to the distance between their languages and English [41–43]. Hindi bilinguals experience a statistically significant penalty of about 4 percentage points, while Hindi late learners earn up to 9 percentage points less than comparable monolinguals. Lastly, Europeans are the only group that earns a premium of about 4 and 6 percentage points for bilinguals and late learners, respectively. We also decomposed the wage gaps for each language group. We found that the coefficient estimates for most groups follow the overall trend, where language skills were more beneficial at the lower end of the earnings distribution, except for Europeans and Hindi.

**Table 2:** Wage regressions by language groups.

	Spanish	Asian	Chinese	Hindi	European	Cohort
Constant	9.064***	8.958***	8.822***	8.891***	8.746***	8.933***
	(0.046)	(0.033)	(0.060)	(0.057)	(0.068)	(0.008)
Bilingual	-0.014***	-0.050***	0.011	-0.039***	0.040***	-0.007***
	(0.002)	(0.006)	(0.010)	(0.013)	(0.005)	(0.002)
Late Learner	-0.037***	-0.064***	-0.048***	-0.088***	0.059***	-0.024***
	(0.002)	(0.004)	(0.007)	(0.009)	(0.004)	(0.002)
Cognitive	0.461***	0.600***	0.653***	0.519***	0.529***	0.498***
	(0.003)	(0.008)	(0.014)	(0.018)	(0.007)	(0.003)
Manual	0.146***	0.258***	0.229***	0.236***	0.197***	0.192***
	(0.003)	(0.008)	(0.012)	(0.016)	(0.007)	(0.003)
Interpersonal	0.191***	0.173***	0.194***	0.178***	0.159***	0.193***
	(0.003)	(0.006)	(0.009)	(0.012)	(0.006)	(0.002)
Hours worked	Yes	Yes	Yes	Yes	Yes	Yes
Industry	Yes	Yes	Yes	Yes	Yes	Yes
Demographics	Yes	Yes	Yes	Yes	Yes	Yes
Location Quot.	Yes	Yes	Yes	Yes	Yes	Yes
*Observations*	487,709	122,667	45,647	27,948	117,633	828,325
*Adjusted R* ^2^	0.457	0.490	0.458	0.529	0.493	0.490

Notes: 1. ***p < .01; **p < .05; *p < .1; 2. This table presents the results of OLS regressions, in which the dependent variable is the logarithm of wages; 3. Standard errors are clustered at the state level; 4. Demographic characteristics include sex, race, marital status, level of education, hours worked per week, and the cohort of arrival in the US; 5. All language subsamples consist of the title language speakers, including both bilinguals and late learners, as well as monolinguals; 6. The Asian subgroup is exclusive of Chinese speakers. 7. Language-specific location quotients are incorporated in the models (e.g., in the Spanish model, the Spanish location quotient is added); 8. The last column reproduces Model 4 in Table 1 but adds a cohort of arrival time trend rather than using this variable in the three-way matching.

The last column in Table 2 checks the robustness of results against the cohort of immigration. We re-matched the samples, excluding the cohort variable from the set of matching covariates and adding it to the regression as a time trend. The estimates did not change, validating the negative effect of being a bilingual or late learner on earnings.

Our research findings hold even when accounting for different time periods (e.g., the decade of arrival) or when examining interactions between different language groups (not reported in this study). It is worth noting that self-reported levels of English proficiency might not perfectly reveal proficiency, as shown in studies about self-reported health status [63]. However, differences between groups are likely as large as other within-group differences, such as the difference between males and females.

## Conclusion

This article is intended to investigate the relationship between heritage bilingualism on earnings and identify complementarities between language and non-language skillsets. Language skills differ from other types of skills in that they are acquired with near-zero time and resource investments during childhood. Overlooking the favorable cost-benefit ratio of early language literacy leads to potential underestimation of the value of language skills and might result in underinvestment [4]. In the literature review, we highlight different transmission mechanisms: the market value of languages, accumulation of non-language skills, and incomplete assimilation of dual language speakers into the majority population.

To test these pathways, we conducted an econometric analysis of a pooled cross-section of three language groups in the US: English monolinguals, bilinguals, and late learners. The distinction between bilinguals and late learners heeds hypotheses concerning critical periods for the acquisition of languages. While there is debate about the nature of critical periods [13], scholars agree that the ability to learn a second language declines with age and does so at an increasing rate. Additionally, to identify non-language skills, we condensed O*NET job task descriptors into three principal components that are interpreted as measures of cognitive, manual, and interpersonal skills. Although our analysis does not permit us to make causal claims, our methodological refinements offer novel insights into the relationship between language and non-language skills, as well as their interaction with expected earnings in the US context.

Early acquisition of language skills may have the potential to mitigate income disparities by providing individuals who lack traditional hard skills with greater employment opportunities. Our results are consistent with prior evidence ruling out language earnings premia in the US economy and alleging greater financial penalties for Spanish and Asian speakers [6]. Our contribution is the finding that language skills might be of greater benefit to lower-income workers and could close a significant part of the bilingual-monolingual wage gap. We are confident that our estimates are lower-bound estimates of the true effect of multiple language proficiency on earnings because language status is a proxy for residual heterogeneity that negatively affects earnings, such as discrimination and lack of social capital.

Distributional analysis conducted using regression decomposition reveals that variations in the effects of language skills are consistent with the nature of work in lower-paying occupations and the lower degree of differentiation of non-language skill inputs. Since early investment in language skills is a very cheap investment without clear opportunity costs, promoting language acquisition and efforts to maintain bilingual heritage in bilingual households are efficient policy prescriptions.

Furthermore, we did not find supporting evidence for dynamic complementarities between language and non-language skills. In particular, it appears as though cognitive capital does not mediate the effects of language skills on earnings. These results are neither conclusive nor comprehensive, yet they are inconsistent with theories of brain development and do not indicate a greater accumulation of cognitive skills among bilingual speakers. The improved executive control functions developed by multilingual individuals are seemingly unrelated to cognitive development, or they might not primarily lead to monetary outcomes [19]. To temper this conclusion, we highlight the non-monetary benefits of being bilingual or actively engaging in language learning [20], ranging from greater life satisfaction to delayed onset of chronic diseases.

## Supporting information

S1 File Supporting information (including supporting methods and supporting tables). (PDF)
