# Supplementary material for: The labor market outcomes of bilinguals in the United States: Accumulation and returns effects
Source: PLoS One. 2023 Jun 29;18(6):e0287711. doi: 10.1371/journal.pone.0287711 (PMC10309611; doi:10.1371/journal.pone.0287711)
Supplement: S1 File — (PDF) [file pone.0287711.s001.pdf]

Supporting information for:
"The Labor Market Outcomes of Bilinguals in the United States: Accumulation and Returns Effects"

Olga Churkina[1,2*], Luísa Nazareno[1], Matteo Zullo[1,2]

1 Andrew Young School of Policy Studies, Georgia State University, Atlanta, Georgia, United States of America
2 School of Public Policy, Georgia Institute of Technology, Atlanta, Georgia, United States of America

¤ All authors contributed equally to this work.
* Correspondence to: ochurkina1@gsu.edu

Theoretical framework

We employ a model where every worker is endowed with a skill bundle of cognitive, manual, interpersonal and language skills $\mathbf{x} = (x_C, x_M, x_I, x_L)$. The three non-language skills are scored on a continuous scale, and language skills are operationalized dichotomously assigning 0 to monolinguals and 1 to bilinguals or late learners.

Based on skill formation literature [1, 2], we assume that language acquisition in earlier stages of development affects the development of other skills through dynamic complementarities. Cognitive, manual, and interpersonal skills at any given time-period can be represented as a function of non-language skills $(x_{C,t-1}, x_{M,t-1}, x_{I,t-1})$ and language skills $x_{L,t-1}$ at the prior time-period:

$$x_{i\in\{C,M,I\},t} = f(x_{i\in\{C,M,I\},t-1}, x_{L,t-1})$$

Language skills have a direct effect on cognitive skill accumulation by enhancing the ability to deal with cognitive uncertainty and have more minor effects on manual and interpersonal skills accumulation [3, 4]. Also, non-cognitive skills affect accumulation of cognitive skills at a later time [2] and not vice versa. Therefore, early language acquisition might impact cognitive development but it might not impact non-cognitive development. At time $t+1$, we have:

$$x_{i\in\{C,M,I\},t+1} = g(x_{C,t}) = g(f(x_{i\in\{C,M,I\},t-1}, x_{L,t-1}))$$

The skill requirements of each job $\mathbf{y} = (y_C, y_M, y_I, y_L)$ govern differences in earnings. The four-dimensional equilibrium assignment resulting from unique matching of workers to jobs is given by:

$$\begin{bmatrix} y_C^* \\ y_M^* \\ y_I^* \\ y_L^* \end{bmatrix} = D_x y^* \begin{bmatrix} x_C \\ x_M \\ x_I \\ x_L \end{bmatrix}$$

where $D_x y^*$ is the Jacobian of the matching function $\mu : \mathbf{x} \to \mathbf{y}$, mapping worker skills into job skill demands [5]. We observe positive assortative matching when workers

match to jobs loading more heavily on the worker's competitive advantage [6], for example when workers with greater cognitive skills (x_C) match to jobs demanding higher levels of cognition (y_C). Conversely, we observe a mismatch when a Jacobian differs from the identity matrix (i.e., $x_i \neq y_i^*$). The wedge between worker skills and skill requirements of their job is represented by their mean squared deviation $E[(y_i - x_i)^2]$; when non-zero, this indicates either underplacement or overplacement of a worker.

Workers with language skills have access to the main monolingual job market and the bilingual job market. In a developed economy, the monolingual market is large in size, and asymptotic properties apply: as the number of jobs j grows larger, worker skills and skill requirements of the job converge (i.e., $E[(y_i - x_i)^2] \to 0$). On the contrary, the bilingual job market is typically undersized and characterized by frequent mismatches (i.e., $E[(y_i - x_i)^2] \neq 0$).

Towards the lower end of the income distribution, coupling language and non-language tasks unlocks economies of scale [7]. Economies of scale secure wage premia for bilingual speakers who would be otherwise substituted by a monolingual worker and translator pair. The more specialized the skills requirements of a job, the stronger the case for decoupling language and non-language. Thus, the model indicates opportunity for 'skill rents' from language proficiency clustered at the lower end of the wage distribution, and little opportunity for skill rents for workers employed in cognitively-intensive occupations in the main monolingual job market. For these workers, language turns into an 'idle skill.'

Sparse principal component analysis

SPCA is a specialized technique used in statistical analysis and, particularly, in the analysis of multivariate data sets. It extends the classic method of principal component analysis (PCA) to reduce data dimensionality by introducing sparsity structures to the input variables. The standard PCA algorithm searches for linear combinations

$$a_k' x \quad (k = 1, ..., p)$$

while capturing maximum variance in the input space, subject to

$$a_k' a_k = 1 \quad \& \quad a_h' a_k = 0 \, (k \geq 2, \, h < k)$$

where a_h and a_k are vectors of coefficients for the h-th and k-th principal components, respectively, $\mathbf{x} = (x_1, ..., x_p)'$ is a vector of input variables with unit variance and R is a correlation matrix.

Given that the principal components are linear combinations of all input variables in ordinary PCA, SPCA overcomes this disadvantage by finding linear combinations containing a limited set of input variables. We implemented the SCoTLASS (Simplified Component Technique — LASSO) approach to parameterize the sparsity [8, 9]. For the j-th element of the vector a_k and a tuning parameter t, the SCoTLASS approach introduces extra constraints

$$\sum_{j=1}^{p} |a_{kj}| \leq t$$

Therefore, $t \geq \sqrt{p}$ reduces down to classical PCA. if $t < 1$, there is no solution; and for $t = 1$ and for each k, there is a unique non-zero loading a_{kj}.

We found sparse loadings that correspond to the weights for each original variable when calculating the principal components and the scores that contain the original data in a rotated coordinate system, following [10]. The main advantage of the SCoTLASS method is the absence of rotation (orthogonal, etc.) that preserves the variance of initially discovered principal components while the loadings are forced to carry extremely small or large values.

S1 Table. Skills from O*NET and factor loadings.

	Loadings		
**Skills (O*NET code)**	Interpersonal	Cognitive	Manual
**Basic Skills**			
Active Learning (b1)	0.583	0.705	
Active Listening (b2)	0.556	0.512	-0.512
Critical Thinking (b3)	0.547	0.745	
Learning Strategies (b4)	0.666	0.523	
Mathematics (b5)		0.716	
Monitoring (b6)	0.713	0.412	
Reading Comprehension (b7)	0.435	0.715	
Science (b8)		0.707	
Speaking (b9)	0.604	0.457	-0.527
Writing (b10)	0.475	0.644	-0.411
**Complex Problem-Solving Skills**			
Complex Problem Solving (c1)	0.537	0.777	
**Resource Management Skills**			
Management of Financial Resources (m1)	0.611		
Management of Material Resources (m2)	0.653		
Management of Personnel Resources (m3)	0.858		
Time Management (m4)	0.801		
**Social Skills**			
Coordination (so1)	0.854		
Instructing (so2)	0.758	0.404	
Negotiation (so3)	0.794		
Persuasion (so4)	0.748		
Service Orientation (so5)	0.656		-0.455
Social Perceptiveness (so6)	0.791		-0.423
**Systems Skills**			
Judgment and Decision Making (sy1)	0.610	0.691	
Systems Analysis (sy2)	0.554	0.744	
Systems Evaluation (sy3)	0.570	0.745	
**Technical Skills**			
Equipment Maintenance (t1)			0.902
Equipment Selection (t2)			0.897
Installation (t3)			0.614
Operation and Control (t4)			0.856
Operation Monitoring (t5)			0.903
Operations Analysis (t6)		0.669	
Programming (t7)		0.744	
Quality Control Analysis (t8)			0.865
Repairing (t9)			0.895
Technology Design (t10)		0.623	
Troubleshooting (t11)			0.957
**SS Loadings**	9.988	8.272	7.837
**Proportion Var**	0.285	0.236	0.224
**Cumulative Var**	0.285	0.522	0.746

Note: *Factors obtained from exploratory factor analysis.*

S2 Table. Initial sample composition by year, in percent.

	Monoling.	Biling.	Types of Biling.					Late L2	Types of Late L2				
			EU	Spanish	Asian	Hindi	Other		EU	Spanish	Asian	Hindi	Other
2005	97.73	0.66	0.11	0.41	0.10	0.02	0.03	1.61	0.23	0.96	0.29	0.05	0.08
2006	97.54	0.70	0.11	0.44	0.10	0.02	0.03	1.76	0.26	1.03	0.32	0.06	0.09
2007	97.48	0.72	0.11	0.46	0.10	0.02	0.03	1.80	0.26	1.09	0.30	0.06	0.09
2008	97.39	0.75	0.11	0.48	0.11	0.02	0.03	1.85	0.27	1.10	0.32	0.06	0.10
2009	97.25	0.81	0.12	0.51	0.12	0.02	0.03	1.94	0.28	1.14	0.34	0.06	0.12
2010	97.10	0.83	0.11	0.54	0.12	0.02	0.04	2.07	0.28	1.24	0.37	0.07	0.11
2011	96.95	0.87	0.12	0.55	0.12	0.02	0.04	2.18	0.28	1.32	0.39	0.08	0.12
2012	96.86	0.90	0.13	0.58	0.12	0.03	0.04	2.24	0.29	1.34	0.40	0.08	0.12
2013	96.75	0.96	0.12	0.62	0.14	0.03	0.05	2.30	0.27	1.41	0.40	0.08	0.13
2014	96.66	1.03	0.13	0.67	0.15	0.03	0.05	2.31	0.28	1.40	0.40	0.09	0.13
2015	96.56	1.11	0.14	0.74	0.15	0.03	0.06	2.33	0.30	1.42	0.39	0.09	0.14
2016	96.51	1.12	0.14	0.75	0.14	0.03	0.05	2.38	0.29	1.42	0.42	0.10	0.15
2017	96.37	1.20	0.15	0.80	0.15	0.04	0.06	2.43	0.29	1.46	0.43	0.10	0.15
2018	96.35	1.22	0.15	0.82	0.16	0.04	0.06	2.43	0.28	1.45	0.43	0.10	0.17
2019	96.27	1.23	0.15	0.81	0.16	0.05	0.07	2.50	0.28	1.51	0.43	0.11	0.18
Total	96.91	0.94	0.13	0.61	0.13	0.03	0.04	2.14	0.28	1.29	0.38	0.08	0.12

Note: *Initial sample: N=12,865,608, including 100,435 bilinguals, 220,998 late learners, and 12,544,175 monolinguals.*

S3 Table. Results of three-way matching.

	Retained Ratio, %		Matched Sample			Initial Sample		
	Bilingual	Late Learner	Bilingual	Late Learner	Monolingual	Bilingual	Late Learner	Monolingual
2005	97.80	93.30	4,541	9,917	24,197	4,643	10,629	825,361
2006	97.97	93.72	5,030	11,459	26,913	5,134	12,227	843,582
2007	97.56	93.69	5,236	12,006	27,486	5,367	12,815	849,503
2008	97.84	94.55	5,576	12,783	29,930	5,699	13,520	865,234
2009	98.70	96.10	5,918	13,383	30,930	5,996	13,926	846,494
2010	98.10	94.34	5,783	13,253	30,684	5,895	14,048	817,116
2011	97.87	93.52	5,686	13,121	30,299	5,810	14,030	792,718
2012	98.22	94.22	6,229	13,850	32,209	6,342	14,699	800,645
2013	98.51	95.33	6,589	14,622	34,581	6,689	15,339	824,538
2014	99.14	96.12	7,008	15,011	36,353	7,069	15,617	824,732
2015	98.58	95.61	7,632	15,174	38,237	7,742	15,870	830,975
2016	98.39	94.64	7,688	15,537	39,240	7,814	16,417	838,027
2017	98.36	95.06	8,395	16,296	41,942	8,535	17,142	853,242
2018	98.35	95.42	8,663	16,476	42,735	8,808	17,267	860,925
2019	99.06	96.93	8,808	16,916	44,665	8,892	17,452	871,083

Note: *Initial sample: N=12,865,608; Matched sample: N=818,987.*

S4 Table. Sample characteristics.

	Bilingual		Late Learner		Monolingual	
	Sample Means	Difference	Sample Means	Difference	Sample Means	Difference
Cognitive	0.555***	0.000	0.522***	0.000	0.560***	0.025***
	(0.001)	(0.001)	(0.001)	(0.001)	(0.000)	(0.000)
Manual	0.375***	-0.000	0.405***	-0.001	0.384***	0.014***
	(0.001)	(0.001)	(0.001)	(0.001)	(0.000)	(0.000)
Interpersonal	0.410***	-0.000	0.405***	-0.001	0.405***	-0.002***
	(0.001)	(0.001)	(0.001)	(0.001)	(0.000)	(0.000)
Male	0.526***	-0.001	0.578***	-0.004**	0.542***	-0.016***
	(0.002)	(0.002)	(0.001)	(0.002)	(0.001)	(0.001)
Female	0.474***	0.001	0.422***	0.004**	0.458***	0.016***
	(0.002)	(0.002)	(0.001)	(0.002)	(0.001)	(0.001)
Age	34.809***	0.168***	34.906***	0.519***	35.493***	8.490***
	(0.039)	(0.055)	(0.026)	(0.036)	(0.019)	(0.019)
White	0.181***	0.003	0.146***	0.003***	0.191***	0.653***
	(0.001)	(0.002)	(0.001)	(0.001)	(0.001)	(0.001)
Black	0.018***	0.001**	0.029***	0.009***	0.031***	0.062***
	(0.000)	(0.001)	(0.000)	(0.001)	(0.000)	(0.000)
Latin	0.614***	-0.007***	0.578***	-0.019***	0.595***	-0.561***
	(0.002)	(0.002)	(0.001)	(0.002)	(0.000)	(0.000)
Asian	0.175***	-0.001	0.238***	0.002	0.172***	-0.163***
	(0.001)	(0.002)	(0.001)	(0.001)	(0.000)	(0.000)
Other race/ethnicity	0.012***	0.003***	0.009***	0.005***	0.012***	0.009***
	(0.000)	(0.001)	(0.000)	(0.000)	(0.000)	(0.000)
Less than high school	0.075***	0.003**	0.134***	0.009***	0.056***	-0.017***
	(0.001)	(0.001)	(0.001)	(0.001)	(0.000)	(0.000)
High school	0.618***	-0.004**	0.590***	-0.010***	0.641***	-0.027***
	(0.002)	(0.002)	(0.001)	(0.002)	(0.001)	(0.001)
College and higher	0.307***	0.002	0.276***	0.001	0.303***	0.045***
	(0.001)	(0.002)	(0.001)	(0.001)	(0.001)	(0.001)
Married	0.468***	-0.004**	0.536***	-0.008***	0.484***	0.112***
	(0.002)	(0.002)	(0.001)	(0.002)	(0.001)	(0.001)
Single	0.438***	-0.002	0.383***	-0.008***	0.424***	-0.178***
	(0.002)	(0.002)	(0.001)	(0.001)	(0.001)	(0.001)
Separated or widowed	0.094***	0.006***	0.082***	0.015***	0.092***	0.066***
	(0.001)	(0.001)	(0.001)	(0.001)	(0.001)	(0.001)

Notes: 1. ***$p < .01$; **$p < .05$; *$p < .1$; *2. Initial sample: N=12,865,608; Matched sample: N=818,987; 3. Standard errors in parentheses; 4. For categorical variables, means are equivalent to sample proportions; 5. Differences are calculated by subtracting the means of the initial sample from the means of the matched sample.*

S5 Table. Quantile wage regressions.

	10 pct.	25 pct.	50 pct.	75 pct.	90 pct.
Constant	6.765***	7.668***	8.849***	10.004***	10.682***
	(0.021)	(0.014)	(0.011)	(0.010)	(0.014)
Bilingual	0.014***	-0.011***	-0.034***	-0.006***	0.005
	(0.004)	(0.003)	(0.003)	(0.003)	(0.004)
L2 Learner	0.021***	-0.016***	-0.042***	-0.033***)	-0.023***
	(0.003)	(0.002)	(0.002)	(0.002)	(0.003)
Cognitive	0.232***	0.434***	0.610***	0.575***	0.602***
	(0.006)	(0.005)	(0.004)	(0.004)	(0.005)
Manual	0.184***	0.207***	0.236***	0.245***	0.140***
	(0.005)	(0.004)	(0.004)	(0.004)	(0.006)
Interpersonal	0.146***	0.207***	0.216***	0.229***	0.233***
	(0.004)	(0.003)	(0.004)	(0.004)	(0.006)
Hours worked	0.031***	0.025***	0.017***	0.012***	0.013***
	(0.0002)	(0.0001)	(0.0001)	(0.0001)	(0.0001)
Industry	Yes	Yes	Yes	Yes	Yes
Demographics	Yes	Yes	Yes	Yes	Yes
Location quotients	Yes	Yes	Yes	Yes	Yes
Year	Yes	Yes	Yes	Yes	Yes
Observations	818,987	818,987	818,987	818,987	818,987
Adjusted R^2	0.204	0.318	0.370	0.302	0.280

Notes: 1. $^{***}p < .01$; $^{**}p < .05$; $^{*}p < .1$*; 2. Standard errors are clustered at the state level; 3. The table reports OLS regressions, in which the dependent variable is the RIF transform of wages; 4. Demographic characteristics include sex, race, marital status, level of education, hours worked per week, and the cohort of arrival in; the US; 5. The regression results correspond to Figure 2 in the main text.*

References

1. Cunha F, Heckman J. The Technology of Skill Formation. American Economic Review. 2007;97(2):31–47.

2. Cunha F, Heckman JJ, Schennach SM. Estimating the Technology of Cognitive and Noncognitive Skill Formation. Econometrica. 2010;78(3):883–931.

3. Bialystok E. Bilingualism: The Good, the Bad, and the Indifferent. Bilingualism: Language and Cognition. 2009;12(1):3–11.

4. DeLuca V, Rothman J, Bialystok E, Pliatsikas C. Redefining Bilingualism as a Spectrum of Experiences that Differentially Affects Brain Structure and Function. Proceedings of the National Academy of Sciences. 2019;116(15):7565–7574.

5. Lindenlaub I. Sorting Multidimensional Types: Theory and Application. The Review of Economic Studies. 2017;84(2):718–789.

6. Postel-Vinay F, Lindenlaub I. Multidimensional Sorting under Random Search. In: 2017 Meeting Papers. 501. Society for Economic Dynamics; 2017.

7. Coombs CK, Cebula RJ. Are there Rewards for Language Skills? Evidence from the Earnings of Registered Nurses. The Social Science Journal. 2010;47(3):659–677.

8. Jolliffe IT, Trendafilov NT, Uddin M. A Modified Principal Component Technique based on the LASSO. Journal of Computational and Graphical Statistics. 2003;12(3):531–547.

9. Zou H, Hastie T, Tibshirani R. Sparse Principal Component Analysis. Journal of Computational and Graphical Statistics. 2006;15(2):265–286.

10. Bartkowiak A, Trendafilov NT. Feature Extraction by the SCoTLASS: An Illustrative Example. In: Intelligent Information Processing and Web Mining. Eds. in Klopotek, M. A., Wierzchen, S. T. and Trojanowski, K. Springer; 2005. p. 3–11.
